# A developmental delay linked missense mutation in Kalirin-7 disrupts protein function and neuronal morphology

**DOI:** 10.3389/fnmol.2022.994513

**Published:** 2022-12-01

**Authors:** Euan Parnell, Roos A. Voorn, M. Dolores Martin-de-Saavedra, Daniel D. Loizzo, Marc Dos Santos, Peter Penzes

**Affiliations:** ^1^Department of Neuroscience, Feinberg School of Medicine, Northwestern University, Chicago, IL, United States; ^2^Department of Biochemistry and Molecular Biology, School of Pharmacy, Instituto Universitario de Investigación en Neuroquímica, Complutense University of Madrid, Madrid, Spain; ^3^Department of Neurology, Feinberg School of Medicine, Northwestern University, Chicago, IL, United States; ^4^Centre for Autism and Neurodevelopment, Feinberg School of Medicine, Northwestern University, Chicago, IL, United States

**Keywords:** developmental delay, neuron, spine, NMDAr, neurodevelopment

## Abstract

The Rac1 guanine exchange factor Kalirin-7 is a key regulator of dendritic spine morphology, LTP and dendritic arborization. Kalirin-7 dysfunction and genetic variation has been extensively linked to various neurodevelopmental and neurodegenerative disorders. Here we characterize a Kalirin-7 missense mutation, glu1577lys (E1577K), identified in a patient with severe developmental delay. The E1577K point mutation is located within the catalytic domain of Kalirin-7, and results in a robust reduction in Kalirin-7 Rac1 Guanosine exchange factor activity. In contrast to wild type Kalirin-7, the E1577K mutant failed to drive dendritic arborization, spine density, NMDAr targeting to, and activity within, spines. Together these results indicate that reduced Rac1-GEF activity as result of E1577K mutation impairs neuroarchitecture, connectivity and NMDAr activity, and is a likely contributor to impaired neurodevelopment in a patient with developmental delay.

## Introduction

Neuronal network function is highly dependent on the dendritic morphology of neurons, which harbor the synaptic connections through which input is received, processed and integrated (Harris and Kater, 1994; Hausser et al., 2000; Alvarez and Sabatini, 2007). Most excitatory synapses are located on dendritic spines: membranous and protein dense protrusions of dendrites (Harris, 1999; Carlisle and Kennedy, 2005; Alvarez and Sabatini, 2007). The formation, stabilization and maintenance of dendritic structure are required for synaptic development and plasticity. Synaptic activity drives morphological changes and can induce long-term potentiation (LTP) and long-term depression (LTD) of synaptic junctions, processes essential to learning and memory (Harris and Kater, 1994; Yuste and Bonhoeffer, 2001). Many developmental and psychiatric disorders display affected structural and functional plasticity, resulting in deficiencies in neural connectivity (Forrest et al., 2018).

Rho family guanosine nucleotide exchange factors (GEFs) are key regulators of dendrite and spine morphogenesis, being highly important in the remodeling of the actin cytoskeleton to drive spine growth and formation (Nakayama et al., 2000; Jaffe and Hall, 2005; Tada and Sheng, 2006; Tolias et al., 2011). GEFs interact with small GTPases to drive GDP for GTP exchange, inducing an active GTP-bound state (Rossman et al., 2005; Bai et al., 2015). This active state is temporally controlled by intrinsic Rho GTPase activity that cleaves GTP to GDP, returning the GTPase to the inactive state. The GTPases Rac1, Cdc42, and RhoA are important factors in shaping the dendritic arbor (Threadgill et al., 1997; Nakayama et al., 2000); Rac1 and Cdc42 promote dendritic branching and the formation, enlargement and maintenance of spines, whereas RhoA decreases and apposes these processes. Kalirin-7, a GEF for Rac1 has been found to be a key player in driving dendritic arborization and dendritic spine morphogenesis through Rac1.

Kalirin-7 has been found to localize to the post synaptic density (PSD) of dendritic spines, and to contribute to spine formation, maturation, LTP and LTD, and dendritic arborization (Parnell et al., 2021). Kalirin-7 activates Rac1, facilitating the interaction and relocalization of downstream effectors, including P21–activated kinase (PAK) that activates remodeling of the actin cytoskeleton and contributes to the formation of spines and dendritic arborization (Johnson et al., 2000; Penzes et al., 2001a,b; Penzes and Remmers, 2012).

The Rac-GEF catalytic domain of Kalirin is composed of a typical Dbl homology (DH)- pleckstrin homology (PH) module (Parnell et al., 2021). Whereas the DH domain directly interacts with Rac1 to drive activity, the PH auxiliary domain has varying function in related GEFs—it can tether enzyme activity to membrane regions via lipid interactions, regulate Rac-GEF activity (Liu et al., 1998; Chhatriwala et al., 2007), and couple with regulatory partners to provide intermolecular regulation of GEF activity (Chakrabarti et al., 2005; Kiraly et al., 2011). Indeed, the Rac1-GEF domain of Kalirin-7 has been shown to directly interact with the NR2B subunit of the NMDAr, via the PH domain (Kiraly et al., 2011; Lemtiri-Chlieh et al., 2011). Kalirin-7 plays a critical role in NMDAr dependent structural plasticity and LTP, by regulating synaptic levels of the NMDAr as well as the α-amino-3-hydroxy-5-methyl-4-isoxazolepropionic acid receptor (AMPAr) (Xie et al., 2007; Ma et al., 2008; Lemtiri-Chlieh et al., 2011; Herring and Nicoll, 2016). Thus, there appears to be an intimate relationship between the Rac1-GEF domain of Kalirin, and NMDAr activity and trafficking, linked not only to Rac1 activation, but Kalirin-7/NR2B interactions.

**FIGURE 1:**
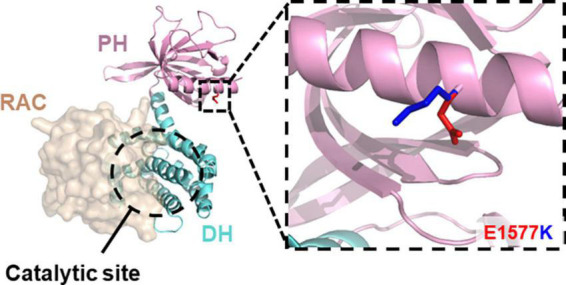
E1577 lies distal to the catalytic GEF site. Homology model of the Kalirin DH- (cyan) PH (pink) in complex with Rac11 (tan, surface) reveals the surface exposed position of glutamate 1577 (inset, blue) and the predicted position of lysine (red) mutant at this site. Note, E1577 lies distal to the catalytic site suggesting indirect modulation of Kalirin GEF activity.

Recent exome sequencing studies [reviewed in Parnell et al. (2021)] have isolated a range of mutations that disrupt Kalirin expression or function and potentially contribute to neurodevelopmental disorder risk, such as ASD (Leblond et al., 2019; Satterstrom et al., 2020), schizophrenia (Kushima et al., 2012; Purcell et al., 2014; Howrigan et al., 2020), and developmental delay/ID (Makrythanasis et al., 2016; Deciphering Developmental Disorders Study, 2017). Moreover, many of these mutations have been predicted or found to disrupt Rac1-GEF activity (Russell et al., 2014), suggesting an integral role for Kalirin Rac1-GEF activity in normal neurodevelopment. A recent, uncharacterized point mutation (DDD4K.02292) 4729G > A, within the Rac1-GEF domain of Kalirin was found in an individual with severe developmental delay (Deciphering Developmental Disorders Study, 2017). This mutation, encoding a single nucleotide polymorphism resulting in glu1577lys substitution (E1577K) within the PH sub-domain, was predicted to be highly deleterious. However, the precise mechanisms through which E1577K impaired Kalirin function was unclear, due to its position distal to the catalytic site (Figure 1). We therefore set out to characterize the functional effects of E1577K mutation on Kalirin function, focusing on the known roles of the PH domain; NMDAr interaction and Rac11-GEF regulation.

E1577K mutation was found to impair the ability of Kalirin-7 to drive dendritic arborization, spine formation and NMDAr-dependent calcium influx within dendritic spines. Interestingly, despite being distal to the catalytic site of Kalirin-7’s Rac1-GEF domain, E1577K mutation was found to ablate Rac1 activation, suggesting an integral role of the auxiliary PH domain in regulating Rac-GEF activity. Moreover, this mutation was found to drastically impair NR2B surface expression and NMDAr activity, suggesting that Rac1-GEF activity may be required for Kalirin-mediated NR2B trafficking. These results suggest a key role for the PH domain in regulating RAC1-GEF activity and provide insight into Kalirin-7-dependent mechanisms contributing to developmental delay.

## Materials and methods

### Homology modeling

Kalirin DHPH model was generated using the Kalirin-7 DH domain (5O33, unpublished) and trio DHPH (6D8Z, Bandekar et al., 2019) crystal structures as templates using Modeler (SaliLab, Webb and Sali, 2021). 5O33 was used to orient Rac relative to the GEF domain of Kalirin-7. All images generated with Pymol2 (Schrodinger).

### Recombinant DNA

PCS2Flag-hKalirin-7 was generated by PCR amplification of human KALRN-7 cDNA (F - GATGAT GATAAGAATATGACGGACCGC/R-TCGAGAGGCCAACTT AACTAAACGTAAGTTGG) and inserted into EcoR1-cut pCS2Flag backbone via ligation independent cloning (Takara Biosciences). The pCS2Flag-hKalirin-7-E1577K construct was generated via site directed mutagenesis, using Quikchange Lightning kit according to manufacturer’s instruction (Agilent), using complementary probes of the sequence; GGAGTGGATCAAGAACATTCGAAAAGTGATTCAAGAAA GGATCATTCACC. pEYFP-NR1a and pEGFP-NR2B were gifts from Stefano Vicini (Addgene plasmids #17928, #17925). pAAV.Syn.GCaMP6f.WPRE.SV40 was a gift from Douglas Kim & GENIE Project (Addgene plasmid # 100837^1^; RRID:Addgene_100837). pEGFP-N2 and 1pmCherry-C1 plasmid was purchased from Clontech (Mountain View, CA, USA). pCS2FLAG was a gift from Peter Klein (Addgene plasmid # 16331^2^; RRID:Addgene_16331).

### Primary neuron culture

Primary cortical cultures were prepared from E18 embryonic Sprague-Dawley rats. Cortices were dissected and homogenized, and cells were dissociated mechanically in papain solution (DNAseI, L-cysteine, EDTA). Cells were strained, counted and plated on poly-D-lysine (PDL) (Sigma-Aldrich) coated 1.5 mm thick, 180 mm diameter coverslips (0.25 μg/coverslip, 2 h) at 2,50,000–3,50,000 cells per coverslip. Neurons were maintained for 1 h in NeuralBasal medium (NBM) (Life Technologies) + 1% B27 (Gibco) + 0.5 mM glutamine + 5% fetal calf serum, before complete media change to growth media; NBM + 1% B27 + 0.5 mM glutamine + 1% Penicillin/Streptomycin. After 4 days in culture, 200 μM D-2-amino-5-phosphonovaleric acid (*D*-APV) was added to growth media. Following maintenance for 3 weeks *in vitro* (WIV), rat neurons were transfected using Lipofectamine 2,000 (Thermo Fisher Scientific) as per the manufacturer’s instructions with indicated constructs.

### Fixation and immunocytochemistry

Neurons were fixed in 3.7% formaldehyde, 4% sucrose in phosphate buffered saline (PBS) at room temperature for 10 min, and washed 3x in PBS at 4°C. For surface staining, anti-NR2B was added directly to live cells and incubated for 30 min before 3x PBS wash and fixation. Coverslips were permeabilized and blocked in PBS + 4% normal goat serum + 0.4% bovine serum albumin + 0.1% TritonX for 30 min, after which all steps were performed in PBS + 2% NGS. Coverslips were incubated with primary antibodies overnight at 4°C, washed 3 times and incubated with secondary antibodies for 1 h at room temperature. Antibodies were diluted in in PBS + 2% NGS. After washing, coverslips were mounted using Prolong Gold (Invitrogen). Antibodies used were against PSD95 (NeuroMab #75028), N2RB (NeuroMab #75-097), vGlut (Synaptic systems #135304) and Kalirin-7 (Penzes et al., 2000).

### Co-immunoprecipitation

Human embryonic kidney 293 cells (Hek293 cells) were grown to confluency in dMEM + 10% FBS in 10 cm dishes. Transfection and co-immunoprecipitation were performed by adjusted protocol (based on Kiraly et al., 2011); Hek293 cells were transfected with Lipofectamine 2000 (Thermo Fisher Scientific), in a ratio of 1 NR1: 3 NR2B: 1.5 KALRN-7/E1577K, with a total amount of 10 μg DNA per 10 cm dish. After transfection, cells were lysed in lysis buffer (50 mM Tris, 100 mM NaCl, 0.1% TritonX100) + complete protease inhibitors (Sigma-Aldrich) by passage through a 28-gauge needle 8 times, followed by centrifugation at 10,000 rpm for 10 min. Equilibrated M2 Flag beads (Sigma) were added to Co-IP samples and incubated for binding overnight at 4°C. After, samples were pelleted and washed 3 times with lysis buffer. Bound proteins were eluted by boiling into Laemmli buffer (1X) for 5 min at 95°C, alongside input samples. Proteins were separated by SDS PAGE, immobilized on PVDF by Western blot, and visualized [Flag M2 (Sigma #F1804), NR2B (Novus Biologicals, #NB100-74475), Kalirin-spectrin (Sigma #02122)].

### Imaging and analysis

Images for morphological dendrite analysis were acquired using a TI2 wide field microscope with CMOS detector (Nikon) at a magnification of 40x (NA 1.00). Large field images were generated from 4 stitched images. Two microgram Z stack images were extracted and were used for the tracing of the arbor in Fiji. Axons were not traced and sholl analysis was performed at a radius of 10 μm on the dendritic arbor.

Images for spine analysis were acquired using a Nikon C2 confocal microscope with C2-DUS PMT detection, at 63× magnification (NA 1.40). Z stacks were acquired spanning the Z depth of the dendritic arbor. Approximately 100 μm of the secondary dendrite was traced for spine analyses. Deconvolution was performed using the deconvolute stack function (NIS elements). Surface NR2B quantification was performed by generating an ROI using GFP channel. This ROI was expanded by 0.5 microns and area measured for normalization purposes. NR2B signal outside of this ROI was cleared, threshold applied and “analyze puncta” was employed to detect puncta larger than 0.2 microns. The same threshold was employed for all images within matched replicates. The number of puncta detected was divided by total dendrite area to control for variation in dendrite area analyzed.

Live cell imaging was performed using a Nikon C2 confocal microscope with Zyla cMOS camera (Andor) at a magnification of 63× at 488 nm, 10 frames per second. For each cell a Z-stack was taken prior to GCAMP6 imaging, to allow tracing of dendritic spines labeled with mCherry cell-fill. Coverslips were transferred to artificial cerebrospinal fluid (aCSF containing in mM: NaCl 125, KCl 2.5, CaCl2 2, glucose 11, NaHCO3 26.2, NaH2PO4 1, HEPES 10, pH 7.4) supplemented with NBQX (5 μM) and TTX (500 nM) to inhibit AMPAr and sodium channels, respectively, allowing visualization of NMDAr-specific calcium influx. Analysis was performed by generating Z scored stacks in FIJI (average intensity *Z*-projection subtracted from each image in stack, and then divided by the standard deviation *Z*-projection) and regions of interest (ROI) were drawn over dendritic spines using the cell-fill red channel. ROI intensity was calculated at each frame and analyzed using custom MATLAB scripts. In brief, a rolling average was employed to reduce photobleaching effects and “find peaks” was employed with a 3x standard deviation filter to identify calcium events within spines. Kalirin-7 overexpression increased baseline noise (Figure 5D), irrespective of baseline fluorescence, suggestive of noise induced by enhanced surface expression (Figure 4A) and relieved magnesium block, resulting in spontaneous opening of NR2B. 3x standard deviation resulted in peak detection of only high amplitude peaks above noise, likely resulting from glutamate release. Spines with no events were omitted and averages per cell were generated based on the calcium event duration, frequency and amplitude.

**FIGURE 2:**
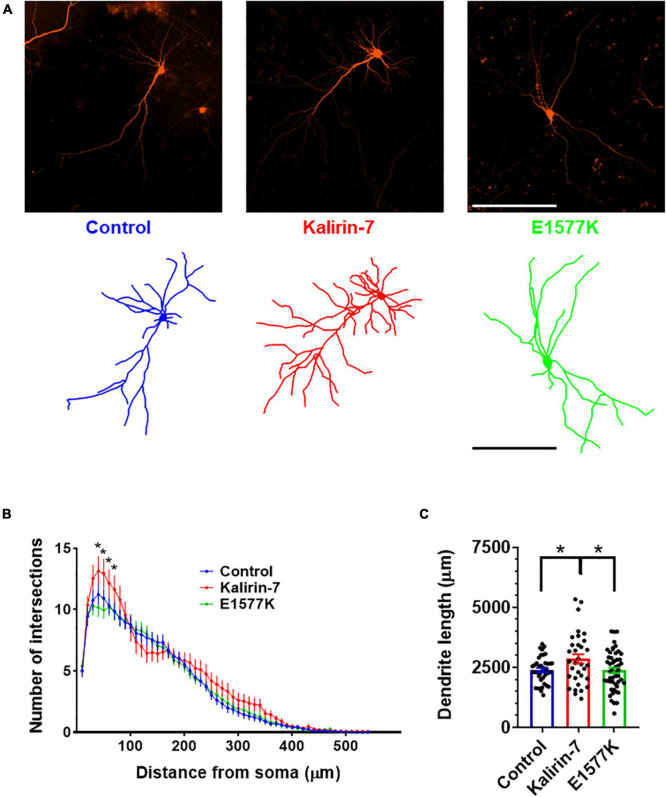
E1577K is deficient in driving dendritic arborization. **(A)** Representative images (Top) and dendritic traces of control (mCherry), Kalirin-7 and Kalirin-7-E1577K (E1577K) transfected rat cultured cortical neurons. Scale bar -200 μm. **(B)** Sholl analysis of dendritic arbors of indicated conditions. **(C)** Average total length of dendritic arbors of indicated conditions. Data points represent average ± SEM, *p < 0.05. *n* = 10–15 neurons per replicate, *N* = 3.

**FIGURE 3:**
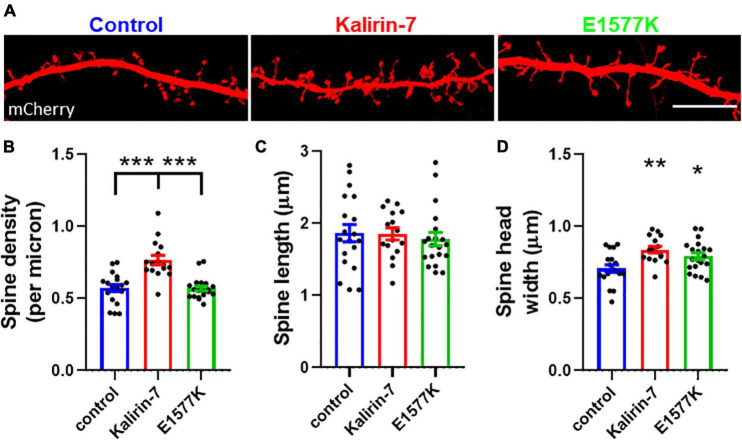
E1577K fails to drive spine formation, but increases spine size. **(A)** Representative regions of mCherry labeled secondary dendrites. Scale bar = 10 μm. **(B)** quantification of spine density per micron of secondary dendrite. Quantification of spine length **(C)** and head width **(D)** per neuron. Data points represent average ± SEM, *p < 0.05. **p < 0.01. ***p < 0.005. *n* = 5–8 neurons per replicate, *N* = 3.

**FIGURE 4:**
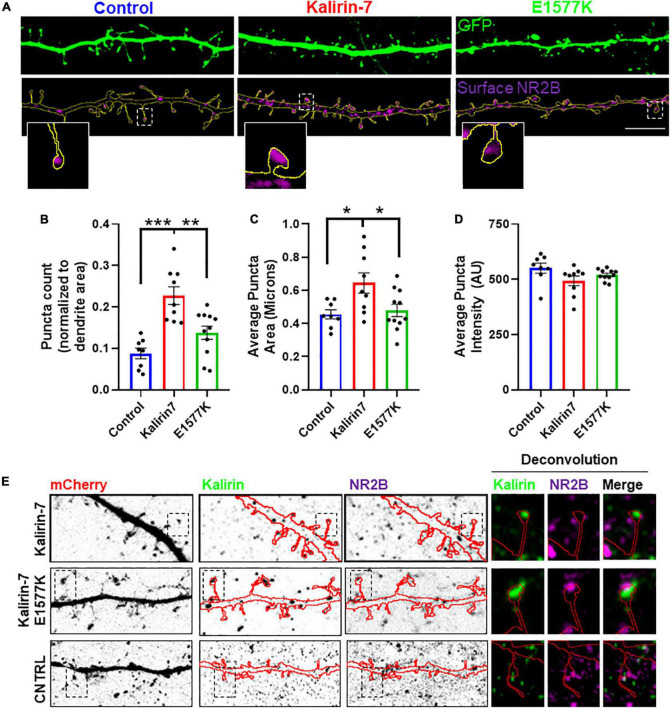
E1577K impairs surface expression of NR2B**. (A)** Representative regions of secondary dendrites with GFP-cell fill and NR2B surface stain. Insets show a single spine from each condition. Scale bar -10 μm. **(B)** Number of NR2B surface puncta normalized to total dendrite (GFP) area measured. **(C)** Average surface NR2B puncta size. **(D)** Average intensity of NR2B surface puncta. *N* = 2, 5 cells per replicate. **(E)** Regions of dendrites stained with Kalirin-7 and total NR2B to assess colocalization following overexpression of indicated constructs. Deconvoluted insets indicate Kalirin-7 and NR2B puncta adjacent within indicated spines. *p < 0.05. **p < 0.01. ***p < 0.005.

**FIGURE 5:**
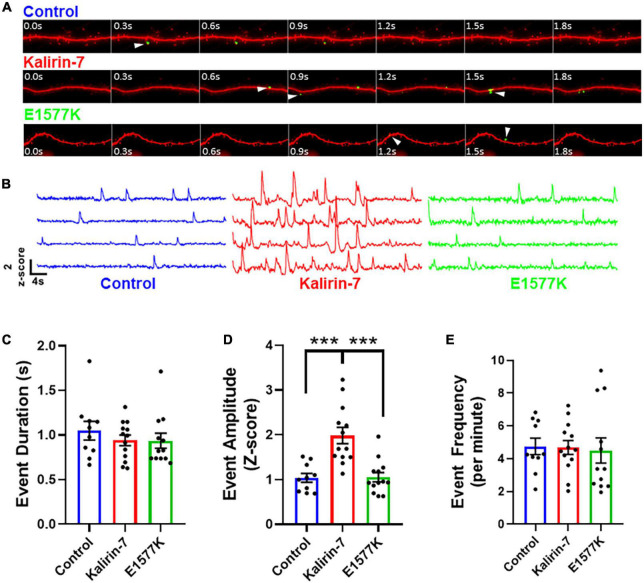
E1577K fails to drive NMDAr-dependent calcium influx. **(A)** 4 s time course of GCamP6 Z-scored live imaging of dendrite with mCherry cell fill (red). Calcium events within spines (white arrowhead) indicate calcium entry through NMDAr channels for each indicated overexpression condition. **(B)** Representative GCamP6 Z-score traces of individual dendrite spines under each overexpression condition (control—blue, Kalirin-7—red, E1577K—green). Quantification of duration **(C)**, amplitude **(D)** and frequency **(E)** of GcamP6 events per individual spine, averaged per cell over 1 min imaging (*n* = 3–4 neurons per replicate, *N* = 3). ***p < 0.005.

### Active-Rac1 pulldown assay

HEK-293T in 10 cm^2^ plates were transfected with Kalirin-7 or E1577K (10 μg) using Lipofectamine 2000 (10 μL), as per manufacturers recommendation. 24 h after transfection, growth medium was replaced with serum-free DMEM for 4 h. Lysis and active Rac1 pulldown was achieved using Peirce Active-Rac1 pulldown kit as per manufacturers instruction; in brief, cells were lysed in 500 μL “lysis/pulldown” buffer with protease inhibitor. Nuclear material was removed by 15-min centrifugation at 15,000xG. Positive and negative samples were prepared by incubation with EDTA (10 mM) and GTP-γ-S (0.1 mM) and GDP (1 mM), respectively, for 30 min, 37°C, before addition of MgCl_2_ (final concentration, 60 mM) to terminate loading. Glutathione Sepharose beads (50 μL slurry per sample), were washed 3x in “Lysis buffer” and coated with GST-PBD (7 μL per sample). Input samples were taken, and 450 μL lysate was applied to PBD-beads, and rotated gently for 1 h at 4°C. Beads were pelleted at 500xG, 30 s, homogenate removed, washed with 500 μL lysis buffer. This was repeated 3 times before addition of 20 μL 2x sample loading buffer, vigorous vortexing and elution of sample at 500xG for 1 min.

### Statistical analyses

Data were analyzed by two-way ANOVA using GraphPad Prism 8 (GraphPad Software Inc.), followed by Tukey’s multiple comparisons post-test for conditions with three independent variables. All experiments were subject to three biological replicates unless otherwise indicated, and cell numbers are indicated in figure legends. Statistical significance *p* < 0.05 (*), *p* < 0.01 (^**^), *p* < 0.005 (^***^) is indicated.

## Results

### E1577K mutation ablates Kalirin-7 mediated dendritic arborization

To assess the functional significance of E1577K mutation, overexpression of mCherry control, mCherry + Kalirin-7 and mCherry + Kalirin-7-E1577K (E1577K) was performed in primary rat cortical cultured neurons. Observations of Kalirin-7 activity have revealed strong alterations in dendritic arborization and spine density/morphology (Russell et al., 2014, 2018; Herring and Nicoll, 2016; Paskus et al., 2019; Grubisha et al., 2021). Neuronal morphology was assessed after 36 h overexpression, after cultures were fixed, stained and dendritic arbors traced (Figure 2A). Sholl analyses revealed a significant increase in proximal dendrite arborization following Kalirin-7 overexpression. However, Kalirin-7-E1577K failed to recapitulate these effects on neuroarchitecture, and arborization was unchanged relative to control neurons (Figure 2B). Overall dendritic length was found to be increased following overexpression of Kalirin-7 (2,387 μm ± 86.1 vs. 2,857 μm ± 188.6, *P* = 0.044), consistent with previous reports (Grubisha et al., 2021) and opposing the effects of Kalirin-7 knockdown (Xie et al., 2010). E1577K failed to induce changes to dendritic outgrowth observed for Kalirin-7 (Figure 2C, 2,857 μm ± 188.6 vs. 2,376 μm ± 115.1, *p* = 0.028).

### E1577K mutation blocks Kalirin-7 mediated dendritic spine formation, but not growth

Spine density and morphological changes have been observed to occur following overexpression of Kalirin-7 (Russell et al., 2014, 2018; Herring and Nicoll, 2016; Paskus et al., 2019; Grubisha et al., 2021). In order to characterize the functional effects on dendritic spine density and morphology, mCherry control, Kalirin-7 WT and E1577K constructs were transfected into cultured rat neurons and ∼100 μm sections of secondary dendrite were imaged for spine assessment (Figure 3A). Kalirin-7 WT produced a robust increase in spine density (0.57 spines/μm ^±^0.024 vs. 0.77 spines/μm ± 0.033, *P* < 0.0001). However, E1577K failed to induce this effect, with spine density indistinguishable from control conditions and significantly reduced as compared to Kalirin-7 overexpression (Figure 3B, 0.77 spines/μm ± 0.033 vs. 0.57 spines/μm ± 0.017, *P* < 0.0001). Neither Kalirin-7 nor E1577K altered dendritic spine length (Figure 3C), however Kalirin-7 overexpression induced a robust alteration in dendritic spine width (0.71 μm ± 0.025 vs. 0.84 μm ± 0.022 microns, *P* = 0.0014) supporting the development of larger, more mature spines. Interestingly, E1577K recapitulated this effect, producing a significant increase in dendritic spine width compared to control (Figure 3D, 0.71 μm ± 0.025 vs. 0.79 μm ± 0.024, *P* = 0.037). Together these results demonstrate that E1577K is unable to drive spinogenesis but retains functionality in driving spine maturation.

### E1577K mutation blocks Kalirin-7 mediated NMDAr surface trafficking

Kalirin-7 has been found to directly interact with and regulate the surface expression of NMDAr receptors, via the NR2B subunit (Kiraly et al., 2011; Lemtiri-Chlieh et al., 2011). As E1577K lies within the PH domain that was found to be the site of NR2B interaction (Kiraly et al., 2011), we set out to assess the effect of Kalirin-7 and E1577K on NR2B trafficking. Neurons were transfected with Kalirin-7 and E1577K, and surface expression of the NR2B receptor was validated (Supplementary Figure 3) and assessed (Figure 4A). Interestingly, Kalirin-7 overexpression resulted in a dramatic increase in the number (Figure 4B, normalized puncta number—0.088 ± 0.13 vs. 0.228 ± 0.21, *P* < 0.0001) and size (Figure 4C, normalized puncta size—0.455 ± 0.028 vs. 0.645 ± 0.061, *P* = 0.0213) of NR2B puncta within transfected neurons, although average NR2B puncta intensity was unchanged (Figure 4D). E1577K expression failed to recapitulate these effects and puncta number and size were significantly reduced compared to Kalirin-7 (normalized puncta number—0.228 ± 0.21 vs. 0.138 ± 0.016, *P* = 0.0018. Average puncta area—0.645 ± 0.061 vs. 0.479 ± 0.037, *P* = 0.0309). No significant difference was observed between control and E1577K for either parameter.

These results suggest that Kalirin-7, but not E1577K, can drive surface expression of NR2B. In order to assess whether this alteration in NR2B trafficking was due to altered localization of mutant Kalirin-7, neurons were stained for total NR2B and Kalirin-7 to assess their subcellular distribution. Both Kalirin-7 and E1577K proteins were found to localize to dendritic spines, colocalizing with PSD95 and with NR2B (Figure 4E), and juxtaposed vGlut (Supplementary Figure 1). These results suggest that both Kalirin-7 and E1577K are directed to the dendritic spine, though E1577K is robustly impaired in its ability to direct NR2B to the surface.

### E1577K mutation blocks Kalirin-7 mediated NMDAr potentiation

In order to prove the functional implications of enhanced NR2B surface expression, we set out to image NMDAr channel activity following overexpression of mCherry control, Kalirin-7-WT and E1577K. Primary cortical neurons were cotransfected with mCherry, GcamP6 (Chen et al., 2013) and either Kalirin-7 or E1577K mutant constructs. Neurons were transferred to artificial cerebrospinal fluid (aCSF) containing NBQX and TTX to inhibit AMPAr and sodium channels, respectively. Thus calcium entry is blocked through calcium-permeable AMPAr, and depolarization is inhibited, blocking calcium entry through voltage gated calcium channels. Together these interventions facilitate the observation of calcium events mediated by NMDAr (Supplementary Videos 1–3). In addition, magnesium was retracted, allowing NMDAr calcium influx independently of depolarization (Figure 5A). ROIs were drawn over individual spines using the mCherry channel, and Z-scored GcamP6 signal was measured at each frame within the ROI, as previously reported (Walker et al., 2017; Figure 5B). Importantly, neuronal activity was blocked completely with *D*-APV (Supplementary Figures 2A,B) and application of NMDA resulted in a robust influx of calcium (Supplementary Figures 2C,D), confirming that NMDAr channel activity specifically was observed. Overexpression of Kalirin-7 and E1577K had no effect on the duration of calcium influx events (Figure 5C), however, Kalirin-7 had a robust effect on the amplitude of NMDAr calcium events (Figure 5B, *Z*-score amplitude—1.037 ± 0.099 vs. 1.983 ± 0.181, *P* = 0.0001) supporting increased NMDAr content within spines, consistent with surface staining (Figure 4). Despite colocalization with NR2B within dendritic spines, E1577K failed to recapitulate this enhancement with NMDAr event amplitude significantly reduced as compared to Kalirin-7 (Z-score amplitude—1.983 ± 0.181 vs. 1.052 ± 0.103, *P* = < 0.0001). No alteration in calcium event frequency was observed for either Kalirin-7 or E1577K overexpression conditions (Figure 5E) as expected, as presynaptic release probability is unlikely to be altered following post-synaptic alterations in Kalirin-7 signaling. These results indicate that Kalirin-7 drives NMDAr surface expression at excitatory synapses to promote their activity within dendritic spines, and that E1577K mutation blocks these effects. Importantly, impaired NMDAr content and activity during neurodevelopment may play a powerful role in the presentation of developmental delay in the affected patient by impairing NMDAr-mediated synaptic plasticity.

### Mutation at E1577 ablates Rac1-GEF catalytic activity

**FIGURE 6:**
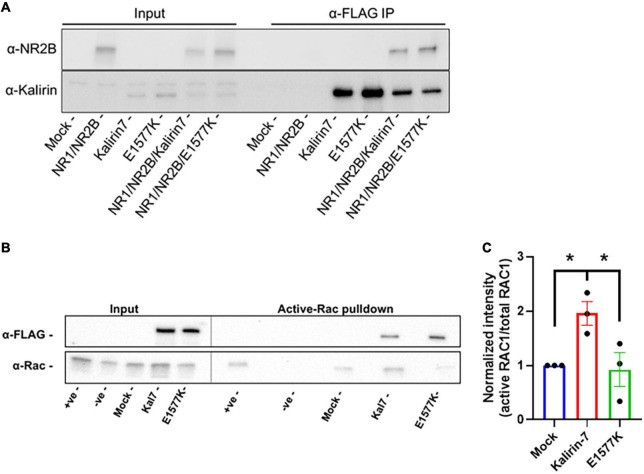
E1577K shows impaired Rac1-GEF activity. **(A)** Western Blot of anti-FLAG pulldown of Kalirin-7 and E1577K from HEK293T, with co-immunoprecipitation of transfected NR2B (Mock = non-transfected). **(B)** HEK293T cells were transfected with the indicated conditions and active-Rac1 was pulled down from lysates with PAK-PBD coated Sepharose beads. Positive (+ ve) and Negative (-ve) samples indicate Mock transfected lysates Loaded with GTP-γ-S and GDP, respectively. Blots were probed with anti-FLAG and anti-Rac1. **(C)** Quantification of Active-RAC1 pulldown under the indicated conditions. *p < 0.05.

E1577K failed to recapitulate the effects of Kalirin-7 on dendritic arborization, spinogenesis and NMDAr activity within dendritic spines. Kalirin-7 is known to directly interact with NR2B, providing a potential mechanism underlying the observed deficit in E1577K-mediated NMDAr trafficking and activity. We therefore set out to assess the interaction strength between Kalirin-7/E1577K by heterologous co-immunoprecipitation. HEK293T were co-transfected with YFP-NR1, GFP-NR2B (Luo et al., 2002) and either Kalirin-7 or E1577K and anti-FLAG pulldown targeting Kalirin-7 recapitulated previous reports of direct Kalirin-7 interaction with NR2B (Figure 6A). The interaction with NR2B was not affected by E1577K mutation, suggesting that other mechanisms may underly the observed deficits in NMDAr trafficking and activity imparted by E1577K mutation. The location of E1577 is distal to the catalytic site (Figure 1) but within the overall catalytic DH-PH domain. Therefore, we hypothesized that E1577K mutation may impair catalytic activity. We therefore assessed Kalirin-7 and E1577K Rac1-GEF activity by heterologous active-Rac1 pulldown. HEK293T were transfected with GFP control, Kalirin-7 and E1577K, before serum starvation and lysis. Active-Rac1 was pulled down with PAK-PBD conjugated to GST-sepharose beads, alongside positive and negative controls (mock transfected cells incubated with GDP or the non-hydrolysable GTP analog, GTP-γ-S, see materials and methods). Kalirin-7 induced a robust increase in the levels of active Rac1 as compared to mock transfection (Figures 6B,C, + 95.8% ± 22%, *P* = 0.0485). However, E1577K failed to raise active-Rac1 levels above baseline, and showed significantly decreased Rac1 activation compared to that of Kalirin-7 WT (-103% ± 31%, *P* = 0.361%), indicating that E1577K is catalytically inactive. Thus, loss of Rac1-GEF activity is impairs the ability of Kalirin-7 to drive NMDAr surface expression and activity, likely contributing to the patient’s observed developmental delay.

## Discussion

The KALRN gene has been linked to neurodevelopmental disorder risk, with mutations in KALRN implicated in schizophrenia (Kushima et al., 2012; Purcell et al., 2014; Russell et al., 2014, 2018; Howrigan et al., 2020), ASD (Leblond et al., 2019; Satterstrom et al., 2020), developmental delay (Deciphering Developmental Disorders Study, 2017), and intellectual disability linked to hereditary homozygous loss of KALRN-expression (Makrythanasis et al., 2016). Here we show that a single point mutation within the Rac1-GEF PH domain of KALRN, found in a patient with developmental delay, results in loss of catalytic activity. Indeed, although Kalirin-7 was able to drive dendritic arborization/growth, spine formation, NMDAr surface expression and activity as previously reported (Penzes et al., 2001a,b; Xie et al., 2007; Lemtiri-Chlieh et al., 2011; Russell et al., 2014; Herring and Nicoll, 2016), the E1577K mutant was deficient in all these functional outcomes of Rac1-GEF activity. These results suggest that Kalirin-7 GEF activity is required for the trafficking and surface expression of NMDAr. It is therefore likely that loss of Kalirin-7 GEF activity in patients harboring the E1577K mutation is a contributing factor to impaired neurodevelopment. Indeed, impaired surface expression and NMDAr activity is likely to have profound impact on activity-dependent synaptic plasticity, supporting KALRN expression and activity as vital to normal neurodevelopment.

Interestingly, the E1577K mutant retained the ability to drive spine morphological changes, with overexpression of the mutant resulting in an increase in spine size similar to wild type. This observation is consistent with loss of Rac1-GEF activity, as the N-terminal region of Kalirin was found to drive spine size increases following truncation of the Rac1-GEF domain (Ma et al., 2014), likely by acting as a scaffold for post-synaptic density and cell adhesion molecules, such as Neuroligan-1 (Paskus et al., 2019).

The observation of a point mutation in the auxiliary PH domain blocking GEF activity supports a role for the auxiliary PH domain in DH-mediated Rac1-GEF activity, as previously reported for related GEF proteins (Liu et al., 1998; Das et al., 2000). Interestingly, phosphorylation at T1590 by CDK5, proximal to the E1577 mutation site, has been shown to drive NMDAr to dendritic spines (Li et al., 2019), suggesting that this region of Kalirin-7 is involved in the regulation NMDAr surface expression. It is tempting to hypothesize that T1590 phosphorylation may act as a regulatory switch, altering Rac1-GEF activity essential to the normal trafficking of NMDAr to the membrane. E1577K, imparting a net negative to positive charge opposite to phosphorylation, may result in an abrogation or blockade of this regulatory mechanism, resulting in the observed lack of GEF activity. Together, these results suggest that mutations with the Rac1-GEF domain of Kalirin likely contribute to developmental delay via impairment of dendritic arborization, dendritic spine formation, NMDAr surface expression and activity.

## Data availability statement

The original contributions presented in this study are included in the article/Supplementary material, further inquiries can be directed to the corresponding author.

## Author contributions

EP and RV wrote the manuscript and carried out all experiments. EP devised the project. MM-d-S, DL, and MD assisted with experimental procedures and analysis. PP oversaw and funded the project. All authors contributed to the article and approved the submitted version.

## Footnotes

1
http://n2t.net/addgene:100837

2
http://n2t.net/addgene:16331

## Supplementary material

Supplementary Figure 1 Kalirin-7 and E1577K localize to PSD-95/V-Glut positive synaptic sites within dendritic spines. Scale bar = 2 μm.

Supplementary Figure 2 D-APV and NMDA block and induce GCamP6 signaling, respectively. **(A)** Standard deviation Z-projections of 30 seconds before and after D-APV (100 μM) application. Spines showing variation associated with calcium influx are indicated (arrowhead). **(B)** Representative dendritic spine Z-scored traces of calcium events before and after application of D-APV (arrowhead) indicate all calcium events are blocked by NMDAr inhibition. **(C)** 1.5 second timecourse of GCamP6 imaging after application of NMDA (100 μM). **(D)** Representative GCamp6 Z-score traces of individual spine ROIs with application of NMDA (arrowhead). Scale bars = 50 μm.

Supplementary Figure 3 NR2B surface stain validation. Neurons were transfected with GFP-NR2B and surface stained with anti-NR2B. Overlap indicates surface GFP-NR2B. Scale bar = 10 μm.

Supplementary Video 1 Representative videos of GCamP6 spine minis. Z-scored signal (green) with cell fill (red), after transfection with mCherry control.

Supplementary Video 2 Representative videos of GCamP6 spine minis. Z-scored signal (green) with cell fill (red), after transfection with Kalirin-7.

Supplementary Video 3 Representative videos of GCamP6 spine minis. Z-scored signal (green) with cell fill (red), after transfection with E1577K.
