# Supplementary figures and images for: A developmental delay linked missense mutation in Kalirin-7 disrupts protein function and neuronal morphology
Source: Front Mol Neurosci. 2022 Dec 1;15:994513. doi: 10.3389/fnmol.2022.994513 (PMC9751355; doi:10.3389/fnmol.2022.994513)

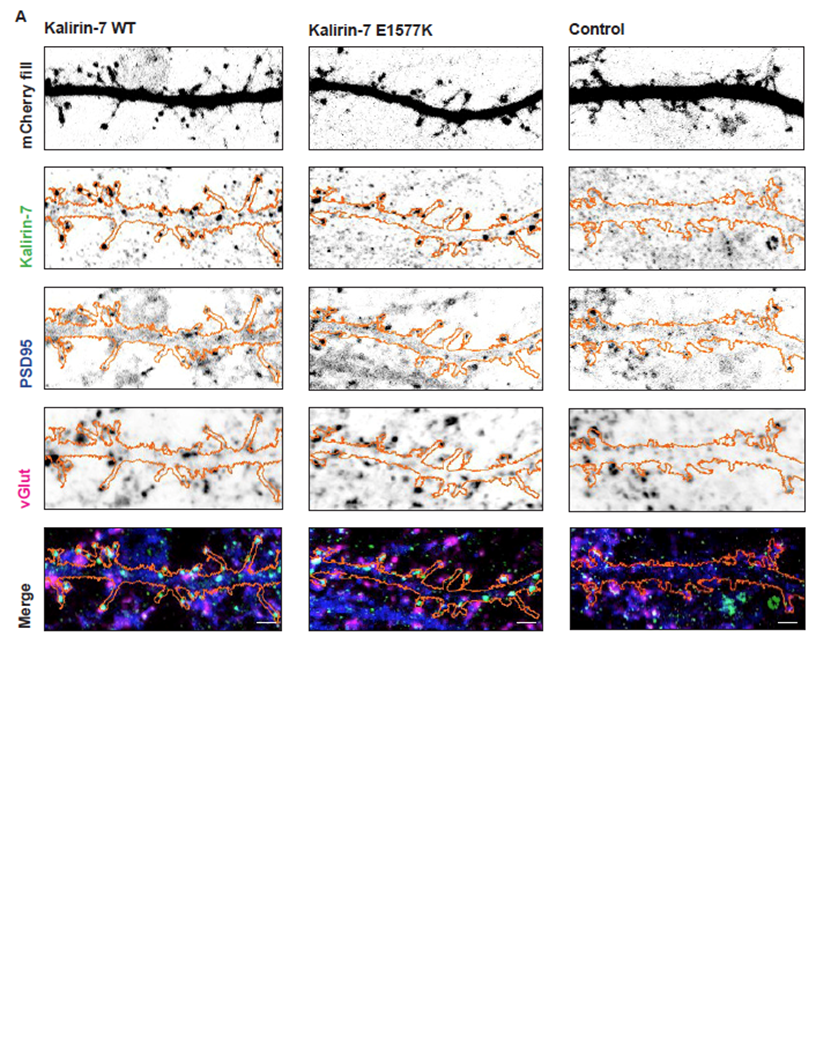

Supplement: Supplementary Figure 1 — Kalirin-7 and E1577K localize to PSD-95/V-Glut positive synaptic sites within dendritic spines. Scale bar = 2 μm. [file Image_1.tif]

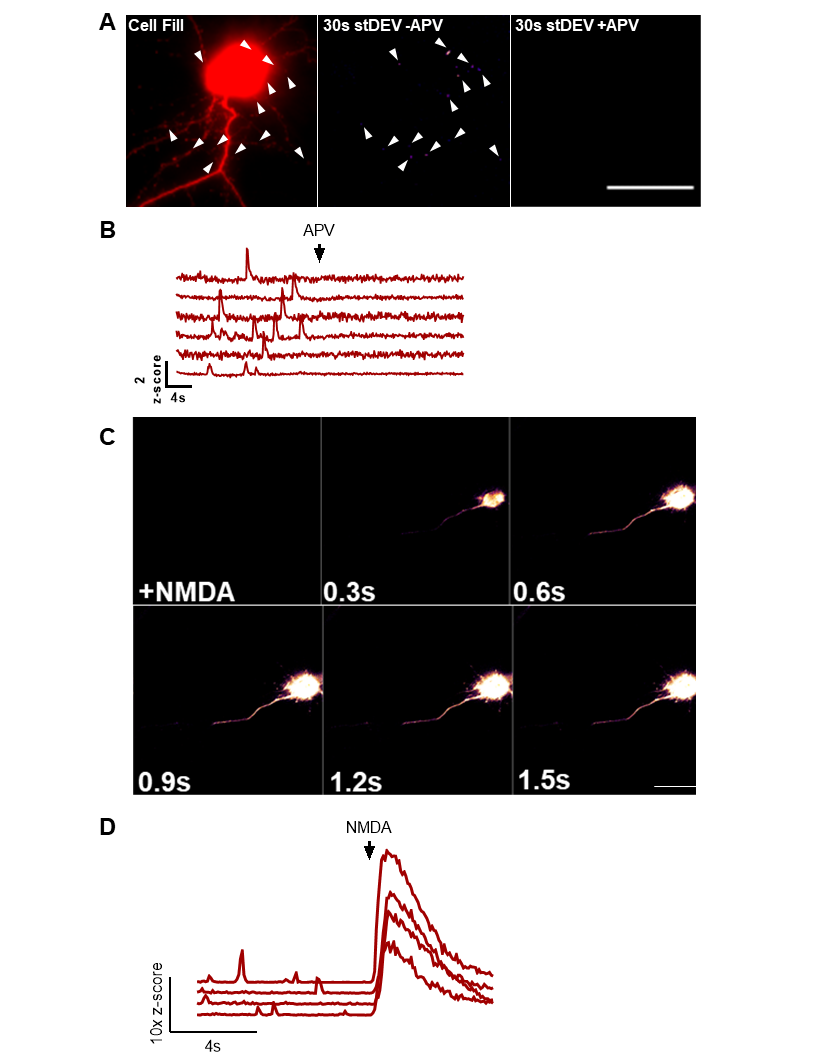

Supplement: Supplementary Figure 2 — D-APV and NMDA block and induce GCamP6 signaling, respectively. (A) Standard deviation Z-projections of 30 seconds before and after D-APV (100 μM) application. Spines showing variation associated with calcium influx are indicated (arrowhead). (B) Representative dendritic spine Z-scored traces of calcium events before and after application of D-APV (arrowhead) indicate all calcium events are blocked by NMDAr inhibition. (C) 1.5 second timecourse of GCamP6 imaging after application of NMDA (100 μM). (D) Representative GCamp6 Z-score traces of individual spine ROIs with application of NMDA (arrowhead). Scale bars = 50 μm. [file Image_2.tif]

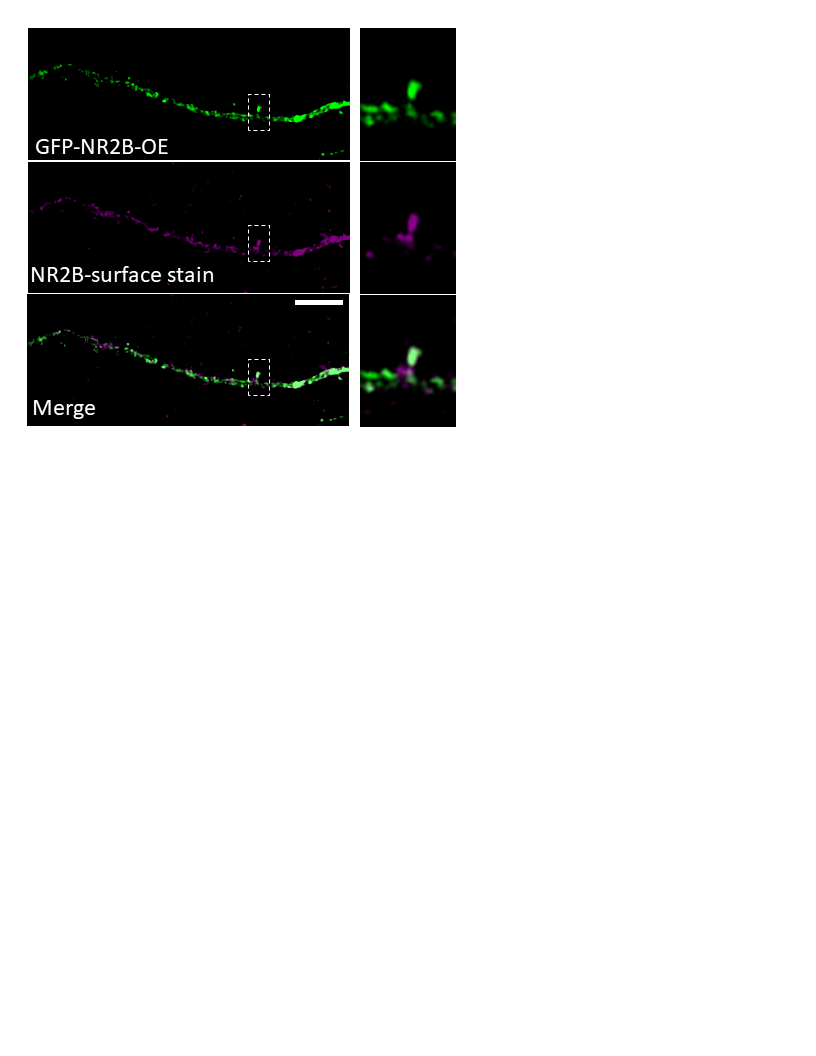

Supplement: Supplementary Figure 3 — NR2B surface stain validation. Neurons were transfected with GFP-NR2B and surface stained with anti-NR2B. Overlap indicates surface GFP-NR2B. Scale bar = 10 μm. [file Image_3.tif]
